# Ultrasonography, Cytology, and Thyroglobulin Measurement Results of Cervical Nodal Metastasis in Patients With Unclear Papillary Thyroid Carcinoma

**DOI:** 10.3389/fendo.2019.00395

**Published:** 2019-06-19

**Authors:** Jong Heon Lim, Dong Wook Kim, Jin Young Park, Yoo Jin Lee, Ha Kyoung Park, Tae Kwun Ha, Do Hun Kim, Soo Jin Jung, Ji Sun Park, Sung Ho Moon, Ki Jung Ahn, Hye Jin Baek

**Affiliations:** ^1^Department of Radiology, Busan Paik Hospital, Inje University College of Medicine, Busan, South Korea; ^2^Department of General Surgery, Busan Paik Hospital, Inje University College of Medicine, Busan, South Korea; ^3^Department of Otorhinolaryngology-Head and Neck Surgery, Busan Paik Hospital, Inje University College of Medicine, Busan, South Korea; ^4^Department of Pathology, Busan Paik Hospital, Inje University College of Medicine, Busan, South Korea; ^5^Department of Nuclear Medicine, Busan Paik Hospital, Inje University College of Medicine, Busan, South Korea; ^6^Department of Anesthesiology and Pain Medicine, Busan Paik Hospital, Inje University College of Medicine, Busan, South Korea; ^7^Department of Radiation Oncology, Busan Paik Hospital, Inje University College of Medicine, Busan, South Korea; ^8^Department of Radiology, Gyeongsang National University Changwon Hospital, Gyeongsang National University School of Medicine, Changwon, South Korea

**Keywords:** papillary thyroid carcinoma, lymph node, metastasis, ultrasonography, fine-needle aspiration, thyroglobulin

## Abstract

**Objective:** This study aimed to evaluate the ultrasonography (US), cytology, and thyroglobulin (Tg) measurement results of nodal metastasis in patients showing unclear US or cytology results of primary papillary thyroid carcinoma (PTC).

**Methods:** From January 2016 to December 2018, 179 patients underwent US-guided fine-needle aspiration (FNA) to diagnose lymphadenopathy in the neck. Among them, 36 patients underwent subsequent total thyroidectomy and nodal dissection, and cervical lymph node (LN) metastasis from PTC was confirmed. However, two patients were excluded because of mismatch between the US and pathological findings of LNs. US images and cytological slides for metastatic LNs were retrospectively analyzed, and serum and FNA Tg levels for metastatic LNs were investigated using data from the electric medical records. Primary PTC patients with suspicious results on both US and cytology were classified as the clear group, and the remaining patients were classified as the unclear group.

**Results:** Of the 34 patients, 24 had clear results of primary PTC on both US and cytology (clear group), whereas 10 had unclear results of primary PTC on US or cytology (unclear group). Of the 10 patients in the unclear group, seven had suspicious nodal metastasis from PTC on cytology after US-guided FNA of the cervical LN, and the remaining three had negative cytology but a positive Tg measurement. Metastatic LNs with cystic change tended to show a positive Tg measurement but negative cytology.

**Conclusions:** The combination of US, cytology, and Tg measurement is necessary for diagnosing nodal metastasis from PTC. In cases with unclear primary PTC on US or cytology, the detection of nodal metastasis may be helpful for assessing primary PTC.

## Introduction

Papillary thyroid carcinoma (PTC) is the most common thyroid malignancy, and most PTC patients have complete removal of the tumor after surgery and/or radioactive iodine treatment (1). However, some PTC patients require additional treatment due to nodal recurrence in the neck (1). These nodal recurrences can be reduced by accurate imaging studies and biopsy before surgery (1, 2). In clinical practice, ultrasonography (US) and US-guided fine-needle aspiration (FNA) are the most widely used tools for detecting nodal metastasis (3, 4). Known suspicious US features of nodal metastasis from PTC include cystic change, calcifications, increased echogenicity, and peripheral or diffuse vascularity (2, 5). However, US diagnosis for nodal metastasis has operator dependency and low sensitivity (6). Moreover, cytological analysis of FNA of the cervical lymph node (LN) may be inadequate or give false-negative results (3). To overcome these limitations, direct measurement of thyroglobulin (Tg) concentration in the needle washout after FNA has been used (3, 7). Although Tg measurement of FNA aspirates is useful for detecting nodal metastasis in PTC patients, its optimal cutoff value is still controversial. Therefore, a combination of these three diagnostic tools is important for detecting nodal metastasis in PTC patients (1, 2).

In some PTC patients, nodal metastasis is suspected on imaging studies or FNA, but the primary thyroid malignancy is unclear (1, 2). When primary thyroid malignancy shows no malignant US features or no malignant cytology, it may be difficult to find the origin of cervical nodal metastasis. In addition, when the primary thyroid malignancy is <2 mm in the largest diameter, it cannot be identified on US. In cases with suspected nodal metastasis in the neck, FNA of the thyroid nodules is recommended regardless of the nodule size (2). Accurate diagnosis of cervical nodal metastasis is helpful for detection of primary PTC and patient management (1, 2). To the best of our knowledge, no previous studies have investigated the US and cytological findings of cervical nodal metastasis in patients with unclear US or cytology results of primary PTC. Therefore, the purpose of this study was to evaluate the US and cytological characteristics of nodal metastasis in patients with unclear US or cytology results of primary PTC.

## Methods

### Patients

This retrospective study was approved by Busan Paik Hospital institutional review board, and the requirement for informed consent was waived. From January 2016 to December 2018, one radiologist (who has performed >50 US-guided FNAs/year for cervical lymphadenopathy over a period of 13 years) performed US-guided FNA to diagnose cervical lymphadenopathy in 179 patients (92 female patients and 87 male patients; mean age: 53.7 ± 20.8 years; age range: 5–90 years). Among them, 36 patients (24 women and 12 men; mean age: 47.1 ± 12.3 years; age range: 22–75 years) underwent total thyroidectomy and nodal dissection, and nodal metastasis from PTC was confirmed after surgery. However, we excluded two patients because of mismatch between US and pathological findings of LNs. Ultimately, 34 patients (23 women and 11 men; mean age: 47.2 ± 12.8 years; age range: 22–75 years) were included.

### Ultrasonographic Examination and Image Analysis

Thyroid or neck US was performed using a high-resolution ultrasound scanner (iU 22, Phillips Medical Systems, Bothell, WA, USA; and Aplio 400, Toshiba Medical Systems, Tokyo, Japan) with a 5–12 MHz or an 8–15 MHz linear probe by the same radiologist. One of the two US instruments was randomly selected for each patient.

In December 2018, one radiologist, who was blinded to the cytology results, investigated the US features of thyroid nodules and cervical LNs by retrospective analysis of US. Each LN was classified as positive or negative according to the respective presence or absence of suspicious US features for nodal metastasis from PTC (including cystic change, microcalcifications, increased echogenicity [focal or diffuse], and abnormal vascularity [peripheral or diffuse]) (2, 5, 8). When a cervical LN showed one or more suspicious US features, it was classified as positive.

### Ultrasonography-Guided FNA and Tg Measurement of Lymph Nodes in the Neck

US-guided FNA was performed by the same radiologist using a free-hand technique, one sampling, and a 23-gauge needle attached to a 5 mL plastic syringe without an aspirator (9). Each sample was smeared onto two to four glass slides, which were immediately alcohol-fixed for Papanicolaou staining. The remaining sample in the syringe was rinsed with normal saline, and 1 mL of the normal saline mixed with aspirate was used for cell blocking and Tg measurement. An electrochemiluminescent immunoassay using the Elecsys automatic system (Roche Modular E170, Mannheim, Germany) was used for measuring Tg levels in the aspirates and serum. When the Tg level of the aspirates was >500 ng/mL or 10-fold higher than the serum Tg level (normal range: 3.5–77 ng/mL), it was classified as positive.

### Cytological Analysis

Cytological slides of nodal metastases in the neck were retrospectively reviewed by one cytopathologist who was blinded to the US and Tg measurement results. When follicular cells showing PTC-like nuclear features, such as nuclear enlargement and overlapping, membrane irregularity showing grooves and pseudoinclusion, and prominent nuclear clearing, were identified within an LN, it was determined as nodal metastasis from PTC.

### Statistical Analysis

When primary PTC was suspected on both US (Korean Thyroid Imaging Reporting and Data system [K-TIRADS] category 5) and cytology (Bethesda category 5 or 6), it was classified as clear. The remaining cases were classified as unclear. The data were tested for normal distribution using a Kolmogorov-Smirnov test. The normal distribution of continuous data was tested with the Kolmogorov-Smirnov test. Normally distributed variables were compared using the independent *t*-test, and expressed as the mean ± SD. Group comparisons of categorical variables were performed using the χ2 test or, for small cell values, Fisher's exact test. All statistical analyses were conducted using SPSS, Version 24.0 (IBM, Armonk, New York, USA). A *p* < 0.05 was considered statistically significant.

## Results

All of the 34 patients underwent total thyroidectomy and neck dissection followed by radioactive iodine ablation. The clinical, US, and cytological findings of the primary PTC cases according to the pre-operative clearness of primary PTC are shown in Table 1. There were significant differences in the K-TIRADS and Bethesda categories of primary PTC between the clear and unclear groups (*p* < 0.0001). However, there was no statistical difference in patient age, gender, tumor location, or BRAF mutation status between the groups (*p* > 0.05). US and cytology results of the metastatic LNs according to the pre-operative clearness of primary PTC are found in Table 2. There was no statistical difference in tumor size, tumor number, Tg measurement result, or cytology result of metastatic LNs between the groups (*p* > 0.05). Additionally, individual US features and levels of metastatic LNs showed no significant differences between the groups (*p* > 0.05).

**Table 1:** Comparison of clinical, ultrasonographic, and cytological characteristics of the 34 patients with primary papillary thyroid carcinoma according to the pre-operative clearness of primary papillary thyroid carcinoma.

**Items**	**Unclear (*n* = 10)**	**Clear (*n* = 24)**	***p-*value**
Age (mean ± SD, year)	48.5 ± 12.3	46.6 ± 13.2	0.698
Gender			0.692
Female	6 (60)	17 (70.8)	
Male	4 (40)	7 (29.2)	
K-TIRADS of primary PTC			<0.0001
2	0	0	
3	0	0	
4	6 (60)	0	
5	4 (40)	24 (100)	
Size of primary PTC (mean ± SD, mm)	10.4 ± 7.2	17.6 ± 13.3	0.117
Location of primary PTC			1.000
Right	7 (70)	18 (75)	
Left	3 (30)	6 (25)	
Isthmus	0	0	
Bethesda category of primary PTC			<0.0001
1	1 (10)	0	
2	2 (20)	0	
3	5 (50)	0	
4	0	0	
5	2 (20)	8 (33.3)	
6	0	16 (66.7)	
BRAF analysis of primary PTC			0.428
Not performed	9 (90)	20 (83.3)	
Negative	1 (10)	1 (12.5)	
Positive	0	3 (4.2)	

*Data are number of items, with percentage in parentheses. SD, standard deviation; K-TIRADS, Korean thyroid imaging reporting and data system; PTC, papillary thyroid carcinoma; US, ultrasonography*.

**Table 2:** Comparison of ultrasonographic and cytopathological findings of the metastatic lymph nodes in the neck according to the pre-operative clearness of primary papillary thyroid carcinoma.

**Items**	**Unclear (*n* = 10)**	**Clear (*n* = 24)**	***p* value**
Size of metastatic node (mean ± SD, mm)	19.2 ± 11.7	17.9 ± 11.5	0.776
US features of metastatic LN			
Cystic change	4 (25)	12 (75)	
Microcalcification	4 (36.4)	7 (63.6)	
Increased echogenicity	6 (35.3)	11 (64.7)	
Abnormal vascularity (peripheral or diffuse)	1 (16.7)	5 (83.3)	
Number of metastatic LN			1.000
Single	0	0	
Multiple	10 (100)	24 (100)	
Location of metastatic LN			0.955
Right	5 (50)	13 (54.2)	
Left	3 (30)	6 (25)	
Both	2 (20)	5 (20.8)	
Level of metastatic LN			
2	3 (30)	7 (70)	
3	8 (30.8)	18 (69.2)	
4	9 (28.1)	23 (71.9)	
5	0	1 (100)	
6	8 (25.8)	23 (74.2)	
Tg measurement of metastatic LN			0.793
Not performed	4 (40)	10 (41.7)	
Negative	0	1 (4.2)	
Positive	6 (60)	13 (54.2)	
Cytological finding of metastatic LN			0.961
No malignancy	3 (30)	7 (29.2)	
Suspicious for nodal metastasis from PTC	7 (70)	17 (70.8)	

*Data are number of items, with percentage in parentheses. SD, standard deviation; US, ultrasonography; LN, lymph node; Tg, thyroglobulin*.

**Figure 1:**
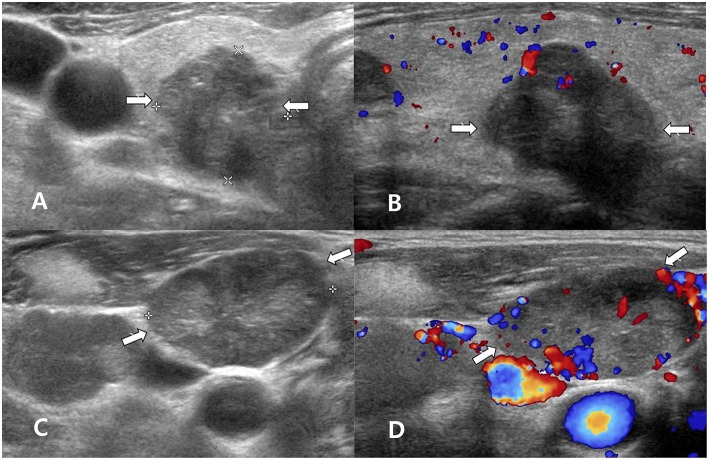
A 60~70 year-old man with clear primary papillary thyroid carcinoma (PTC) on both ultrasonography (US) and cytology. Transverse gray-scale **(A)** and longitudinal color Doppler **(B)** sonography showed a solid thyroid nodule with microcalcifications and a non-parallel orientation in the right thyroid lobe (arrows, 1.8 × 1.8 × 2.1 cm; Korean Thyroid Imaging Reporting and Data system category 5). After US-guided fine-needle aspiration (FNA) of the right thyroid nodule, US-guided FNA was performed for a suspicious lymph node in the right neck, but thyroglobulin measurement was not performed. Transverse gray-scale **(C)** and color Doppler **(D)** sonography showed a suspicious lymph node with increased echogenicity and peripheral vascularity in the right neck (arrows, 2.7 cm in length). In cytology for the right thyroid nodule and lymph node, Bethesda category VI and nodal metastasis from PTC were confirmed, respectively. After total thyroidectomy and both neck dissection, multiple PTCs (classic type) in both thyroid lobes and multiple nodal metastasis from PTC in both necks were confirmed.

**Figure 2:**
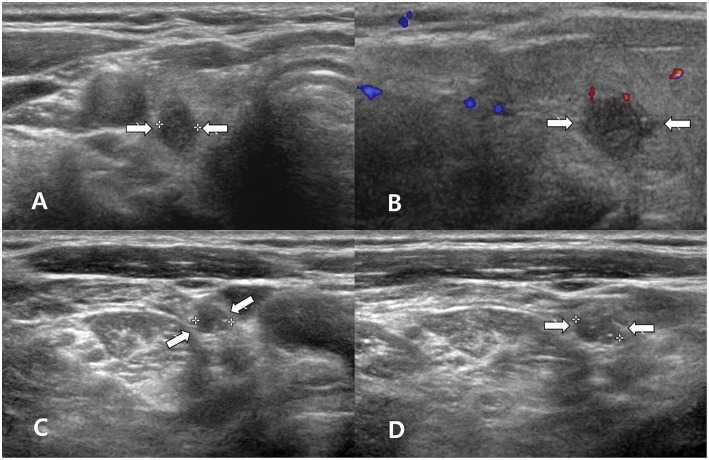
A 60~70 year-old woman with clear primary papillary thyroid carcinoma (PTC) on both ultrasonography (US) and cytology. Transverse gray-scale **(A)** and longitudinal color Doppler **(B)** sonography showed a solid thyroid nodule with spiculated margins and a non-parallel orientation in the right thyroid lobe (arrows, 0.5 × 0.8 × 0.9 cm; Korean Thyroid Imaging Reporting and Data system category 5). After US-guided fine-needle aspiration (FNA) of the right thyroid nodule, US-guided FNA for a suspicious lymph node in the right neck was performed; thyroglobulin measurement was also performed. Transverse **(C)** and longitudinal gray-scale **(D)** sonography showed a suspicious lymph node with increased echogenicity and diffuse vascularity in the right neck (arrows, 0.6 cm in length). Cytology for the right thyroid nodule indicated Bethesda category VI, but there was no malignancy in the lymph node by cytology and thyroglobulin measurement. After total thyroidectomy and right neck dissection, two PTCs (classic type) in the right thyroid lobe and multiple nodal metastasis from PTC in the right neck were confirmed.

Of the 34 patients, 24 (17 women and seven men; mean age: 46.6 ± 13.2 years; age range: 22–75 years) had clear results of primary PTC (mean size: 17.6 ± 13.3 mm, size range: 3.1–51.8 mm) on both US and cytology. Among them, 9 (37.5%) had nodal metastasis from PTC on cytology; a representative example is shown in Figure 1. Of the 24 patients with clear US and cytology results for primary PTC, 14 underwent Tg measurement of the aspirate after US-guided FNA of the cervical LNs, and only one patient (7.1%) had a false-negative result (Figure 2).

**Figure 3:**
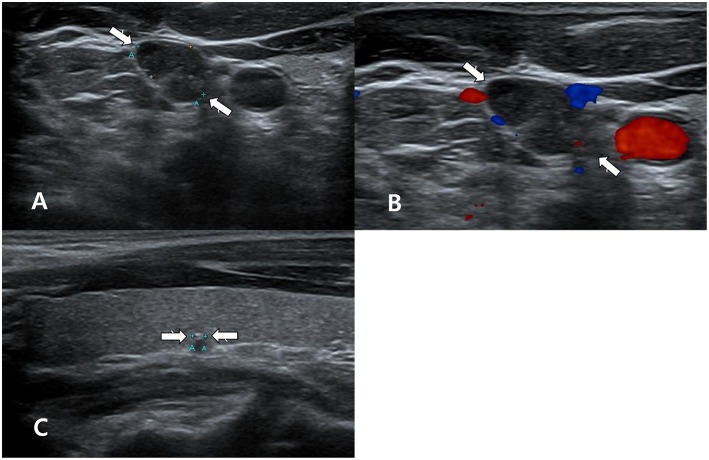
A 40~50 year-old man with unclear primary papillary thyroid carcinoma (PTC) on ultrasonography (US). In the thyroid ultrasound (US), a single suspicious lymph node was found in the right neck **(A,B)**, whereas there was no US abnormality of the thyroid gland, except for a small calcified thyroid nodule in the right lobe (arrow, 0.2 cm in length; Korean Thyroid Imaging Reporting and Data system category 4) **(C)**. Transverse gray-scale **(A)** and color Doppler **(B)** sonography showed a suspicious lymph node with increased echogenicity and microcalcifications in the right lower neck (arrows, 1.3 cm in length). US-guided fine-needle aspiration (US-FNA) with thyroglobulin (Tg) measurement for this lymph node was performed. However, US-FNA for the right calcified thyroid nodule was not performed owing to its small size and calcification. Nodal metastasis from PTC was suspected by cytology and because the Tg level of the FNA aspirates was >500 ng/mL. After total thyroidectomy and right neck dissection, a small PTC (follicular variant, infiltrative type) in the right lobe and multiple nodal metastasis from PTC in the right neck were confirmed.

**Figure 4:**
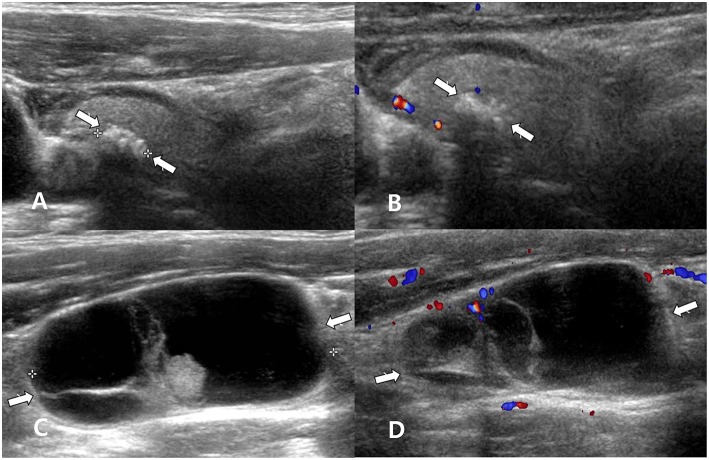
A 50~60 year-old man with unclear primary papillary thyroid carcinoma (PTC) on cytology. Longitudinal gray-scale **(A)** and longitudinal color Doppler **(B)** sonography showed a calcified thyroid nodule with profuse microcalcifications in the right thyroid lobe (arrows, 0.4 × 0.7 × 0.7 cm; Korean Thyroid Imaging Reporting and Data system category 5). Longitudinal gray-scale **(C)** and color Doppler **(D)** sonography showed a suspicious lymph node with cystic change in the right lower neck (arrows, 4.1 cm in length). After ultrasound (US)-guided fine-needle aspiration (FNA) of the right thyroid nodule, US-guided FNA with thyroglobulin (Tg) measurement for a suspicious lymph node in the right neck was performed. The cytological results of the right thyroid nodule and right lymph node revealed no malignancy. However, nodal metastasis from PTC was suspected because the Tg level of the FNA aspirates was >500 ng/mL. After total thyroidectomy and right neck dissection, a PTC (encapsulated variant) in the right lobe and two metastatic lymph nodes from PTC in the right neck were confirmed.

Of the 34 patients, 10 (six women and four men; mean age: 48.5 ± 12.3 years; age range: 33–69 years) showed unclear results of primary PTC (mean size: 10.4 ± 7.2 mm, size range: 1.7–25.6 mm) on US or cytology. One patient had a solitary small calcified thyroid nodule without suspicious US features (Figure 3). In US diagnosis for primary PTC before US-FNA, six patients had K-TIRADS category 4 and four patients had K-TIRADS category 5. In cytological analysis for primary PTC after US-guided FNA, Bethesda categories 1 (*n* = 1), 2 (*n* = 2), 3 (*n* = 5), and 5 (*n* = 2), were observed; there were no patients with Bethesda categories 4 or 6. Of the 10 patients in the unclear group, seven had suspicious nodal metastasis from PTC in cytology after US-guided FNA of the cervical LNs. The remaining three had negative cytology but positive Tg measurement; a representative case is shown in Figure 4.

Of the 34 patients, 19 (55.9%) underwent Tg measurement of FNA aspirates after US-guided FNA of metastatic LNs. US diagnosis, cytology, and Tg measurement of the metastatic LNs in these patients according to the pre-operative clearness or unclearness of primary PTC are summarized in Table 3. However, there was no significant difference between the two groups (*p* = 0.896). The number of patients with positive results in all three tests and those with positive US and Tg measurement but negative cytology results were similar. Metastatic LNs with cystic change tended to be positive by Tg measurement but negative by cytology.

**Table 3:** Comparison of ultrasonographic and cytology characteristics and thyroglobulin measurement of the metastatic lymph nodes in the neck from papillary thyroid carcinoma (15 patients in whom thyroglobulin measurement of aspirates after fine-needle aspiration of lymph nodes were not perform were excluded).

**Items**	**Unclear (*n* = 6)**	**Clear (*n* = 13)**
only US (+)	0	1 (7.7)
only cytology (+)	0	0
only Tg (+)	0	0
US and cytology (+), but Tg (–)	0	0
US and Tg (+), but cytology (–)	3 (50)	5 (38.5)
Cytology and Tg (+), but US (–)	0	0
US, cytology, and Tg (+)	3 (50)	7 (53.8)
US, cytology, and Tg (–)	0	0

*Data are number of items, with percentage in parentheses. US, ultrasonography; Tg, thyroglobulin*.

## Discussion

The frequency of nodal metastasis in the neck from PTC has been reported to be as high as 60–70%, and nodal metastasis from PTC is known to be associated with locoregional recurrence (2, 10). Therefore, precise ultrasonographic evaluation of cervical LNs is essential whenever thyroid nodules are detected (1). In clinical practice, the pre-operative detection of nodal metastasis in PTC patients helps surgeons to plan total thyroidectomy and nodal dissection (1). Additionally, accurate pre-operative detection of nodal metastasis in PTC patients is important for managing patient treatment and preventing locoregional tumor recurrence (1, 11).

US, cytology, and Tg measurement have been widely used for detection of cervical nodal metastasis from PTC (1, 2, 11). US has been established as the primary imaging modality for the assessment of LNs in patients with thyroid nodules and proven thyroid cancer (2). The US criteria for suspicious LNs are highly specific and predictive of nodal metastasis (~80–90%) (2, 12, 13). However, US has several limitations, such as low sensitivity and operator dependency (2). Cytology after US-guided FNA of the cervical LNs has high specificity, but a 5–10% inadequacy rate and a 6–50% false-negative rate, especially in LNs with small size or cystic change (3, 14–16). In our study, 10 patients (29.4%, 10/34) had false-negative cytology, and among them, 9 (90%) showed cystic change of the LNs on US. In cases of cystic nodal metastasis from PTC, Tg measurement may be a valuable adjunct to cytology (3, 7, 16, 17). The addition of Tg measurement to cytology increases the detection of nodal metastasis from PTC by 13% (16). However, the cutoff value of FNA Tg has not been unanimously established, ranging from 0.2 to 50 ng/mL (15). Therefore, we believe that the combination of US, cytology after US-guided FNA, and Tg measurements can improve the detection of nodal metastasis, which will help improve patient outcome.

It is well-known that nodal metastasis in the neck may be the initial manifestation of occult thyroid cancer (13, 18, 19). Occult thyroid carcinoma with cervical nodal metastasis has been reported to occur in approximately one-fifth of all cases of thyroid carcinoma (13, 19). Moreover, cystic nodal metastasis can be the first manifestation of disease in cases of occult primary PTC (13, 18, 19). We believe that accurate diagnosis of nodal metastasis leads to not only the identification of hidden primary PTC but also the clarification of unclear primary PTC. In 10 cases with an unclear result of primary PTC on US or cytology in our study, the detection of nodal metastasis was helpful for assessing primary PTC. To clarify this issue, further studies are required.

Nodule size is not a predictor of thyroid malignancy anymore (20). However, nodule size is known to be associated with diagnostic accuracy of US or cytology; the smaller the nodule, the less accurate (21, 22). So, US-guided FNA for thyroid nodules <5 mm is not recommended (1, 2). In the present study, the size of unclear primary PTC group was smaller than that of clear primary PTC group, although a statistical difference in nodule size between two groups was not found.

This study has several limitations. First, the sample size was small. Second, all patients underwent US and cytology for detecting nodal metastasis, whereas some patients did not undergo Tg measurement. Third, a direct correlation between US and histopathologic diagnoses of metastatic LNs was not achieved at the node-by-node or level-by-level basis. Finally, a limited number of US images was used in the evaluation of US features of LNs, as all US images were retrospectively analyzed.

In conclusion, the combination of US, cytology, and Tg measurement is necessary for diagnosing nodal metastasis from PTC. In cases with unclear primary PTC on US or cytology, the detection of nodal metastasis may be helpful for assessing primary PTC.

## Ethics Statement

All procedures performed in studies involving human participants were in accordance with the ethical standards of the institutional and/or national research committee and with the 1964 Helsinki declaration and its later amendments or comparable ethical standards.

## Author Contributions

DWK: concept and design and review of final manuscript. JL and DWK: acquisition of data and manuscript writing. All authors: literature review and refinement of manuscript. HB and DWK: analysis and interpretation of data.

### Conflict of Interest Statement

The authors declare that the research was conducted in the absence of any commercial or financial relationships that could be construed as a potential conflict of interest.
